# Performance of TaqMan probes for the detection of sexually transmitted infections in South African women

**DOI:** 10.4102/ajlm.v10i1.1124

**Published:** 2021-03-31

**Authors:** Nireshni Mitchev, Ravesh Singh, Nigel Garrett, Veron Ramsuran, Abraham J. Niehaus, Koleka P. Mlisana

**Affiliations:** 1 Department of Medical Microbiology, School of Laboratory Medicine and Medical Sciences, University of KwaZulu-Natal, Durban, South Africa; 2 Department of Medical Microbiology, National Health Laboratory Service, Durban, South Africa; 3 Centre for the AIDS Programme of Research in South Africa, Durban, South Africa; 4 Discipline of Public Health Medicine, School of Nursing and Public Health, University of KwaZulu-Natal, Durban, South Africa; 5 KwaZulu-Natal Research Innovation and Sequencing Platform, School of Laboratory Medicine and Medical Sciences, University of KwaZulu-Natal, Durban, South Africa; 6 Department of Academic Affairs, Research and Quality Assurance, National Health Laboratory Service, Durban, South Africa

**Keywords:** sexually transmitted infections, vaginal discharge syndrome, molecular diagnostics, validation, TaqMan

## Abstract

*Neisseria gonorrhoeae, Chlamydia trachomatis, Trichomonas vaginalis* and *Mycoplasma genitalium* are the four main aetiologies of sexually transmitted infections responsible for vaginal discharge syndrome (VDS). Commercially available multiplex polymerase chain reaction (PCR) assays are expensive and generally not customisable. We evaluated a highly customisable singleplex PCR approach by testing it in parallel with the Anyplex™ II STI-7 detection assay in a cohort of South African women that presented with VDS between May 2016 and January 2017. Our multiple singleplex PCR strategy proved to be a simple, accurate, rapid, affordable and scalable option for diagnosing VDS.

## Introduction

The World Health Organization estimates that approximately one million curable sexually transmitted infections (STIs) occur globally each day.^1^ *Neisseria gonorrhoeae, Chlamydia trachomatis* and *Trichomonas vaginalis* are the three most common pathogens causing STIs worldwide, with co-infection also common.^1,2^ Globally, as of 2016, there were approximately 86 million cases of gonorrhoea, 127 million cases of chlamydia and 156 million cases of trichomoniasis among 15–49 year-olds, with estimated prevalence rates of 0.9%, 3.8% and 5.3% in women specifically.^1,3^ Such estimates are important for the effective prevention and control of STIs but are generally lacking for most low- and middle-income countries.^4^ Torrone et al. recently published a meta-analysis of data from African countries that highlights this point.^4^ Prevalence figures varied widely between studies and ranged from 1.4% to 15.2% for *N. gonorrhoeae,* 1.2% to 20.6% for *C. trachomatis* and 6.6% to 29.7% for *T. vaginalis*.^4^ The prevalence of most STIs is generally greater in certain high-risk populations such as those with a high prevalence of HIV co-infection and women aged 15–24 years.^4^

Sexually transmitted infections and their complications are one of the top five reasons why females in low- and middle-income countries attend healthcare facilities.^1,2^ Although the majority of STIs are asymptomatic, asymptomatic STIs can increase the probability of transmission of HIV and other STIs, as well as have adverse effects on reproduction potential and maternal and newborn health.^1,2,5,6^

Syndromic management of STIs is widely practised and was introduced in South Africa in 1995.^7,8^ Unfortunately, due to its inability to effectively identify and treat a large number of individuals with asymptomatic infections, syndromic management has failed to decrease the prevalence of gonorrhoea and chlamydia in South Africa.^7,8^

Diagnosing the aetiology of most STIs using culture methods is notoriously difficult and can take several days to complete. Multiplex polymerase chain reaction (PCR) assays are currently the preferred diagnostic method used for identifying and subsequently managing both symptomatic and asymptomatic STIs in many well-resourced countries.^9^ Since PCR assays are not dependent on organism viability, they are often up to 20% – 30% more sensitive than conventional phenotypic methods and they can simultaneously detect multiple pathogens and also serve as point-of-care tests.^9,10,11,12,13^ Polymerase chain reaction also performs well on noninvasively obtained specimens like urine and self-collected vaginal swabs.^10^

Our study aimed to evaluate the suitability of a highly customisable singleplex real-time PCR approach using commercially available TaqMan® probes (Thermo Fisher Scientific, Waltham, Massachusetts, United States) for the identification of *N. gonorrhoeae, C. trachomatis, T. vaginalis* and *Myocoplasma genitalium* in vaginal samples from our local population. The Anyplex™ II STI-7 detection assay (Seegene, Seoul, South Korea) was used as the comparator method. This widely used multiplex real-time PCR assay displays excellent sensitivity and specificity characteristics compared with other diagnostic tools.^10^

## Methods

### Ethical considerations

Women attending the Prince Cyril Zulu Communicable Disease Centre (Durban, South Africa) for STI care were approached and, if willing to participate, provided written informed consent for the study. Study data were collected, anonymised and managed using password-protected REDCap electronic data capture tools (Vanderbilt University, Nashville, Tennessee, United States), and stored on a secure server. This study was approved by the Biomedical Research Ethics Committee of the University of KwaZulu-Natal (study approval number: BE534/16).

### Samples

As part of the Centre for the AIDS Programme of Research in South Africa 083 cohort study between May 2016 and January 2017, which was previously described in detail, women aged 18–40 presenting for STI care at a clinic in Durban were assessed for enrolment and participation.^7,14^ HIV-positive women, pregnant women and those engaging in sex work were excluded due to predetermined eligibility criteria.^15^ Participants consented to vaginal swab specimen collection for molecular testing. ESwab™ collection and transport kits (Copan Diagnostics, Brescia, Italy) were used as per the manufacturer’s instructions.

### DNA extraction and polymerase chain reaction

Collection swabs were vortexed for 30 s while inside their transport tubes, whereafter 500 *µ*L of the suspension was added to a 1.5 mL Eppendorf tube. This was followed by centrifugation and resuspension of the sediment in 200 *µ*L distilled water. After heating and sonication steps, 5 µL of the resultant sample was added to 15 *µ*L pre-aliquoted master mix. For the TaqMan® probe assays, master mixes were prepared separately for each single target reaction in a 96-well plate. The Anyplex™ II STI-7 detection assay was used according to the manufacturer’s specifications and was performed using a CFX96 real-time PCR system (BioRad, Hercules, California, United States).^10^ TaqMan® probes were used in a singleplex format on the ABI® 7500 real-time PCR instrument from Applied Biosystems (Thermo Fisher Scientific, Waltham, Massachusetts, United States).

### Data management and analysis

Anyplex™ II STI-7 detection assay results were analysed and interpreted with Seegene Viewer software (Seegene, Seoul, South Korea). Contingency 2 × 2 tables were used to determine the sensitivity, specificity, positive predictive value and negative predictive value for each of the TaqMan® probes with Anyplex™ II STI-7 detection assay as the gold standard.^10,16,17^

## Results

A total of 267 women were screened for STIs at an urban clinic in Durban, South Africa. Vaginal discharge (*n* = 106; 39.7%) was the most common symptom reported.^15^ Vaginal swabs were available for molecular investigation from 250 (93.6%) women. Two molecular assays were used in parallel to detect the presence of four sexually transmitted microorganisms implicated in vaginal discharge syndrome.

The yield obtained from 250 samples by each of the two methods ranged between 3.6% and 13.6% (Table 1). At least one of *N. gonorrhoeae, C. trachomatis, T. vaginalis* or *M. genitalium* was identified in 22.8% (57/250) of the study population. *C. trachomatis* was the most common organism found in co-infections and was present in 42% (5/12) of the *N. gonorrhoeae*, 25% (3/12) of the *M. genitalium* and 11% (1/9) of the *T. vaginalis* infections.

**TABLE 1:** Yield obtained with Anyplex™ II STI-7 detection assay and TaqMan® probes, from 250 vaginal swab specimens collected from patients attending Prince Cyril Zulu Communicable Disease Centre (Durban, South Africa) between May 2016 and January 2017.

Detection technique	*N. gonorrhoeae*		*C. trachomatis*		*T. vaginalis*		*M. genitalium*	
	*n*	%	*n*	%	*n*	%	*n*	%
Anyplex II STI-7	12	4.8	34	13.6	9	3.6	12	4.8
TaqMan probes	11	4.4	33	13.2	10	4.0	14	5.6

TaqMan® probe sensitivity ranged from 91.67% to 100%, and specificity ranged from 98.74% to 100.00% (Table 2). Negative predictive values ranged from 99.08% to 100.00% and positive predictive values ranged from 90.00% to 100.00%, except for *M. genitalium* (78.57%).

**TABLE 2:** Performance characteristics of TaqMan® probes for 250 vaginal swab specimens collected from patients attending Prince Cyril Zulu Communicable Disease Centre (Durban, South Africa) between May 2016 and January 2017.

TaqMan probes	Sensitivity (%)	Specificity (%)	Positive predictive value (%)	Negative predictive value (%)
*N*. *gonorrhoeae*	91.67	100.00	100.00	99.58
*C*. *trachomatis*	94.12	99.54	96.97	99.08
*T*. *vaginalis*	100.00	99.59	90.00	100.00
*M*. *genitalium*	91.67	98.74	78.57	99.58

Final results were available after 130 min and 180 min with the TaqMan® probes and Anyplex™ II STI-7 detection assay, respectively. This includes 45 min for DNA extraction and 15 min for results analysis.

## Discussion

The prevalence of any of the main curable STIs, including syphilis and those caused by *N. gonorrhoeae, C. trachomatis* and *T. vaginalis*, in women in KwaZulu-Natal, South Africa was previously reported to be as high as 13%.^18^ In our study, we observed the overall prevalence of STIs to be 22.8%. Our prevalence data is not a true representation of the general population, because we recruited participants from a sexually active cohort of women that presented with symptoms to an STI clinic. The Xpert® CT/NG assay (Cepheid, Sunnyvale, California, United States) was previously used to investigate this cohort of samples.^7^ For the present study, we employed the Anyplex™ II STI-7 detection assay as the comparator, because the Xpert® CT/NG assay can identify *C. trachomatis* and *N. gonorrhoeae*, but not *T. vaginalis* and *M. genitalium*.

Sensitivity, specificity, positive predictive value and negative predictive value characteristics of the TaqMan® probes for the four investigated organisms compared well to the chosen reference method. The only exception was the low positive predictive value observed for *M. genitalium*. This deviation may be due to the small sample size and requires further investigation.

Except for certain antibiotic-resistant organisms, the STIs detected in our study can usually be treated with a course of antibiotics; however, if they remain undiagnosed and subsequently untreated, a range of serious health issues may follow. Complications include elevated HIV transmission rates, infertility as well as life-threatening ectopic pregnancy and stillbirths.^19^ A rapid, accurate and affordable STI diagnostic tool is therefore essential to identify and treat affected individuals.^13^

Molecular testing methods are advantageous over phenotypic testing methods due to the rapid availability of results. Polymerase chain reaction methodologies can also detect various predefined targets at the same time, including drug-resistance determinants. Singleplex PCR methods offer some important advantages over multiplex methods, principally in terms of ease of optimisation, customisability and target quantification. New gene targets of interest can be added in a flexible way without the requirement of a full revalidation process of the existing targets. Our multiple singleplex PCR system can easily be adapted to diagnose various other clinical syndromes caused by bacterial, viral, fungal and parasitic microorganisms, including meningitis, blood stream infections, respiratory infections, diarrhoea, and urinary tract infections. Singleplex PCR systems allow target quantification, which has potential diagnostic, prognostic and clinical monitoring functions.

Turnaround times with our multiple singleplex PCR were 50 min shorter than with the multiplex method. The clinical impact of this time difference, when non-immediately life-threatening infections are concerned, is probably insignificant.

Multiplex PCR methodologies are generally significantly more cost-effective than singleplex PCRs. However, this does not hold true when small numbers of molecular targets are considered.^20^ Costing of the four targets used in our testing system indicates a considerable cost saving of $15.27 (United States dollars [USD]) per test, over the comparator method that is priced at $24.67 USD per test. Affordability can be increased even further by using a 384-well reaction plate, making it an attractive option for high-throughput environments. With our system, we can easily adopt a workflow that is suitable for both research and clinical laboratories.

### Limitations

Limitations of this study include the relatively small sample size and the low positivity rate obtained by both methodologies. Multiple other microorganisms and dual infections may also have been responsible for vaginal discharge syndrome in some women, but were not considered in this investigation.

### Conclusion

The performance of four TaqMan® probes (Thermo Scientific) was assessed by comparison with the Anyplex II STI-7 detection assay. Our multiple singleplex real-time TaqMan® PCR approach proved to be highly sensitive and specific for the detection of *C. trachomatis, N. gonorrhoeae, T. vaginalis* and *M. genitalium*. This approach offers an accurate, cost-effective and scalable option for identifying these pathogens in our patient population.
